# Development and external validation of a clinical prediction model for MRSA carriage at hospital admission in Southeast Lower Saxony, Germany

**DOI:** 10.1038/s41598-020-75094-6

**Published:** 2020-10-22

**Authors:** Gabriele Raschpichler, Heike Raupach-Rosin, Manas K. Akmatov, Stefanie Castell, Nicole Rübsamen, Birgit Feier, Sebastian Szkopek, Wilfried Bautsch, Rafael Mikolajczyk, André Karch

**Affiliations:** 1 Department of Epidemiology, Helmholtz Centre for Infection Research (HZI), Brunswick, Germany; 2 Central Research Institute of Ambulatory Health Care in Germany (ZI), Berlin, Germany; 3 Institute of Epidemiology and Social Medicine, University of Münster, Münster, Germany; 4 Central Laboratory, Klinikum Wolfsburg, Wolfsburg, Germany; 5 Institute for Microbiology, Immunology and Hospital Hygiene, Städtisches Klinikum Braunschweig gGmbH, Brunswick, Germany; 6 Institute for Medical Epidemiology, Biometry, and Informatics (IMEBI), Medical Faculty of the Martin Luther University Halle-Wittenberg, Halle (Saale), Germany; 7 Hanover Medical School, Hanover, Germany

**Keywords:** Policy and public health in microbiology, Infectious-disease diagnostics, Epidemiology

## Abstract

In countries with low endemic Methicillin-resistant *Staphylococcus aureus* (MRSA) prevalence, identification of risk groups at hospital admission is considered more cost-effective than universal MRSA screening. Predictive statistical models support the selection of suitable stratification factors for effective screening programs. Currently, there are no universal guidelines in Germany for MRSA screening. Instead, a list of criteria is available from the Commission for Hospital Hygiene and Infection Prevention (KRINKO) based on which local strategies should be adopted. We developed and externally validated a model for individual prediction of MRSA carriage at hospital admission in the region of Southeast Lower Saxony based on two prospective studies with universal screening in Braunschweig (n = 2065) and Wolfsburg (n = 461). Logistic regression was used for model development. The final model (simplified to an unweighted score) included history of MRSA carriage, care dependency and cancer treatment. In the external validation dataset, the score showed a sensitivity of 78.4% (95% CI: 64.7–88.7%), and a specificity of 70.3% (95% CI: 65.0–75.2%). Of all admitted patients, 25.4% had to be screened if the score was applied. A model based on KRINKO criteria showed similar sensitivity but lower specificity, leading to a considerably higher proportion of patients to be screened (49.5%).

## Introduction

Several studies confirmed that infections with Methicillin-resistant *Staphylococcus aureus* (MRSA) are associated with increased morbidity and mortality^1–3^ as well as with high treatment and consecutive costs^4^. While universal screening at hospital admission is recommended only if MRSA prevalence is high^5^, targeted screening of risk groups was shown to be cost-effective in intermediate-prevalence countries^2,5–8^. Predictive models for the identification of MRSA carriers can contribute to optimizing screening strategies in hospitals, which have to balance sensitivity against the costs and efforts of a higher proportion of persons to be screened^9^.

For Germany, an intermediate-prevalence country, the DIMDI (German Institute for Medical Documentation and Information) recommends selective MRSA screening of patients at risk^10^. However, there are no clear guidelines on how to select these patients. The Commission for Hospital Hygiene and Infection Prevention (KRINKO) at the Robert Koch Institute released in 2008 a list of 11 factors indicating an increased risk of MRSA colonization at hospital admission^11^. In 2014, the list was slightly revised, and contained now 10 risk factors, some of which have to appear in combination; a definite screening recommendation has not yet been available^12^. Using a complex list of risk factors is, however, hardly applicable in everyday clinical practice^13^. The development of a regional screening strategy as proposed by KRINKO^12^ could, thus, be a sensible approach.

This study aimed at the development and external validation of a predictive model for MRSA carriage at hospital admission in the region of Southeast Lower Saxony. The proposed model should translate into a regional screening recommendation that is easy to use in everyday clinical practice.

## Materials and methods

### Study design and population

This study was conducted in the catchment area of the “Hygienenetzwerk Südostniedersachsen”, a cooperation of healthcare providers in eight municipalities in Southeast Lower Saxony to combat hygiene-relevant pathogens. For the construction of the training dataset, we performed a universal screening of newly admitted patients in two hospitals in Braunschweig (with a total of four locations, covering the majority of the city’s population) over 2 weeks. This dataset was used to determine admission prevalence of MRSA carriage and relevant risk factors. All patients aged 18 or above who were admitted between the 18th of November and the 2nd of December 2013 were asked to complete a self-administered questionnaire on the day of admission. Completed questionnaires were collected by study staff; patients could receive assistance in answering the questionnaire if needed. Patients unable to consent (e.g., due to language barriers or consciousness level) could be represented by a next of kin or had to be excluded.

For external validation, a temporally and spatially independent study was performed. For this study, all patients testing positive for MRSA at admission to Wolfsburg hospital (universal screening in place, hospital located as well in the catchment area) between the 7th of September 2015 and the 9th of March 2016 who met the inclusion criteria received the questionnaire; MRSA-negative participants were recruited from all inpatients on two separate days within this period using the same questionnaire as well as the same inclusion and exclusion criteria.

### Questionnaire

The selection of the risk factors examined in the questionnaire was based on the list of “Risk populations for colonization with MRSA” published by KRINKO in 2008^11^. Further variables were included based on literature search and expert opinion. In addition, data on age, sex, education, and occupation were collected. In total, 34 variables per patient (representing potential risk factors) were examined.

### Laboratory analyses

To identify MRSA carrier status, a combined nasopharyngeal swab and an additional swab of chronic wounds (if present) were taken within 48 h after admission. No additional swabs (e.g. from devices) were taken. In the case of multiple inpatient admissions during the study period, only the first admission was evaluated. MRSA was determined by cultivation on selective media. Confirmation of the species (e.g., by catalase plus coagulase) was followed by confirmation of Oxacillin resistance by a second independent method (resistance gene determination by PCR or VITEK 2).

### Data management and statistical analysis

Questionnaire data were read in automatically using TeleForm (Cardiff Software, Vista, California 92081, USA), were continuously monitored and validated, and were linked individually with the results of MRSA screening. In the main analysis, we used simple imputation for missing information on single disease statuses in the questionnaire (imputing missing values as not having had the respective disease).

Groups of patients with positive and negative MRSA status were compared using univariable analyses. χ^2^ tests or Fisher’s exact tests were used for categorical variables. The Wilcoxon rank-sum test was used for the continuous variable age.

Using multivariable logistic regression analysis, we developed a model to predict MRSA carrier status (dependent variable) at the time of hospital admission. Following the Hosmer–Lemeshow suggestion, variables with *p* < 0.25 in univariable analyses were included in the multivariable analysis^13^. Logistic regression with backward selection was performed using fractional polynomials for age as implemented in the Stata function “mfp” to allow for non-linear effects (*p* < 0.2 for selecting polynomials instead of linear effects). A *p* value of ≥ 0.05 (based on Wald tests) was selected as parameter exclusion criterion in the backward selection.

For the final model, a probability cut-off for an optimal balance of sensitivity and specificity was determined by Youden index; the corresponding AUC (area under the receiver operating characteristic (ROC) curve) was calculated. Bootstrap sampling (1.000 replications) was applied to assess stability of variable selection and the effect estimates of the predictive model.

Additionally, a simplified score based on the predictive factors of the model was derived. The derived score was then applied to the validation dataset; subsequently, sensitivity, specificity, and positive and negative predictive values (PPV and NPV) were calculated. Calibration in the validation dataset was assessed by regressing MRSA status on the log odds of the predictor probabilities, which were calculated using the logistic regression model derived in the training dataset^14^. A calibration curve, showing the observed proportions of MRSA-positivity stratified by quantiles of predictions with at least 60 patients per group, was used to investigate goodness of fit among the whole range of predictions (R package ‘rms’^15^ version 5.1-3.1). The model and score were also compared to a model representing the KRINKO risk factors. As there was no question on history of contact with a known MRSA carrier, the KRINKO score had to be constructed without this criterion.

Statistical analyses were performed with Stata IC 12.1 (StataCorp, College Station, US) and R version 3.6.1 (www.R-project.org).

### Sensitivity analyses

We performed an additional univariable analysis with non-imputed variables as a complete case analysis. Since the number of MRSA-positive patients in the training dataset was considerably lower than expected a priori, we also performed a sensitivity analysis in which we used the validation dataset for model development and the training dataset for validation to evaluate if the small number of cases in the training dataset might have affected our analyses.

### Ethics and informed consent

This study received ethics approval from the Ethics Committee of Hanover Medical School (ethics approval reference number 1980–2013 and amendments). All research was performed in accordance with relevant guidelines and regulations. All study participants and/or their legal representatives provided written informed consent.

## Results

### Baseline characteristics in the training dataset

Within the recruitment period, 2556 patients were admitted, and 2065 MRSA swabs (80.8%) were obtained. Thirty-eight percent of all admitted patients (n = 973) did not provide informed consent (neither themselves nor their legal representatives). During data processing, another 382 individuals (14.9%) were found not to meet the inclusion criteria. Finally, 1201 participants (47.0%) were included in the analysis.

Of the 2065 persons tested, 42 participants were MRSA-positive, resulting in a prevalence of 2.0% (95% confidence interval [CI]: 1.5–2.7%).

### Univariable risk factor analysis

Out of 1201 participants who could be included in further analyses, 16 were MRSA-positive (1.3%; 95% CI: 0.8–2.2%) (Table 1). MRSA carriers had a median age of 74 (interquartile range: 59–89) years, and were older than MRSA-negative participants (median 63; interquartile range 45–74; *p* = 0.008).

**Table 1:** Baseline characteristics of the study population (n = 1201) in the training dataset, stratified by MRSA status.

	Total		MRSA status				*p* value
			Positive		Negative		
	N	%	N	%	N	%	
	1201	100	16	1.3	1185	98.7	
Age [years; *median (interquartile range)*]	63.0 (18–97)		73.5 (39–94)		63.0 (18–97)		0.008
Sex							0.541
Male	566	48.7	9	56.3	557	48.6	
Female	597	51.3	7	43.8	590	51.4	
Missing	38		0		38		
Educational level*							0.488
Low	255	22.4	5	31.3	250	22.2	
Medium	617	54.1	9	56.3	608	54.0	
High	269	23.6	2	12.5	267	23.7	
Missing	60		0		60		
Long-term care facility	65	5.4	2	12.5	63	5.3	0.207
Heard about MRSA	527	43.9	8	50.0	519	43.8	0.619
MRSA history	14	1.2	4	25.0	10	0.8	< 0.001
Decolonization attempt	9	0.8	3	18.8	6	0.5	< 0.001
MRSA in the household	10	0.8	0	0.0	10	0.8	0.712
MRSA currently known	5	0.4	1	6.3	4	0.3	< 0.001
Diabetes mellitus	189	15.7	3	18.8	186	15.7	0.739
Any medical diabetes treatment	158	13.1	1	6.3	157	13.2	0.415
Currently under dialysis	16	1.3	0	0.0	16	1.4	0.640
Dialysis in the past	7	0.6	0	0.0	7	0.6	0.758
Chronic skin disease	90	7.5	2	12.5	88	7.4	0.444
Inflammatory bowel disease	47	3.9	1	6.3	46	3.9	0.628
Under cancer treatment	186	15.5	7	43.8	179	15.1	0.002
Care dependency	93	7.7	6	37.5	87	7.3	< 0.001
Burn injury	8	0.7	0	0.0	8	0.7	0.742
Open chronic wounds	46	3.8	2	12.5	44	3.7	0.069
Abscess/purulent skin disease	69	5.8	0	0.0	69	5.8	0.320
Outpatient treatment abroad past 12 months	40	3.3	0	0.0	40	3.4	0.455
Inpatient treatment past 12 months	526	43.8	9	56.3	517	43.6	0.312
Contact to MRSA	5	0.4	0	0.0	5	0.4	0.795
Urinary catheter currently	121	10.1	4	25.0	117	9.9	0.046
Urinary catheter past 6 months	135	11.2	5	31.3	130	11.0	0.011
Antibiotics currently	103	8.6	5	31.3	98	8.3	0.001
Antibiotics past 6 months	369	30.7	9	56.3	360	30.4	0.026
Surgery past 12 months	336	28.0	6	37.5	330	27.9	0.393
Organ transplantation	17	1.4	1	6.3	16	1.4	0.099
Occupational contact with livestock	26	2.2	0	0.0	26	2.2	0.549
Pets	351	29.2	4	25.0	347	29.3	0.708
Work in hospital or practice	52	4.3	0	0.0	52	4.4	0.392
Work in nursing home	26	2.2	1	6.3	25	2.1	0.258
Working in meat processing	7	0.6	0	0.0	7	0.6	0.758

The calculation of proportions does not include missing values in the denominator.

*According to the International Standard Classification of Education (ISCED).

A larger proportion of MRSA-positive than MRSA-negative persons reported being under cancer treatment (43.8% vs. 15.1%, *p* = 0.002), care dependency (care in nursing home or additional care at home) (37.5% vs. 7.3%, *p* < 0.001), a urinary catheter currently administered (25.0% vs. 9.9%, *p* = 0.046) or administered within the past 6 months (31.3% vs. 11.0%, *p* = 0.011), having received any antibiotics during the past 6 months (56.3% vs. 30.4%, *p* = 0.026) as well as more than one class of antibiotics (31.3% vs. 8.3%, *p* = 0.001).

MRSA-positive patients had heard more often of MRSA (50.0% vs. 43.8%, *p* = 0.619), reported more often an MRSA history (25.0% vs. 0.8%, *p* < 0.001), and a decolonization attempt (18.8% vs. 0.5%, *p* < 0.001), and considered themselves more often a current MRSA carrier (6.3% vs. 0.3%, *p* < 0.001). 526 participants (43.8%) had been hospitalized within the past 12 months, only ten of them abroad (< 0.1%); all of them were MRSA-negative.

### Model building

The variables age as well as “MRSA history”, “decolonization attempt”, “MRSA currently known”, “under cancer treatment”, “care dependency ”, “urinary catheter currently administered”, “urinary catheter in the past 6 months”, “antibiotic treatment in the past 6 months” and “various antibiotics in the past 6 months” were considered as potential predictors for further model building based on the univariable results.

We excluded the variables “MRSA currently known” and “decolonization attempt” due to high collinearity with “MRSA history”. After backward selection, the variables “MRSA history”, “care dependency” and “under cancer treatment” remained as predictors in the final model (Table 2). The model predicted MRSA-positivity with a sensitivity of 75.0% (95% CI: 47.6–92.7%) and a specificity of 78.8% (95% CI: 76.4–81.1%) at a probability cut-off of 0.01 determined by Youden index in the training dataset. The PPV was 4.6% (95% CI: 3.4–6.1%) and the NPV was 99.6% (95% CI: 99.0–99.8%). AUC was 0.81 (95% CI: 0.68–0.93), with 78.8% of all patients being correctly classified.

**Table 2:** Final multivariable model (n = 1201).

	OR (95% CI)	*p* value
MRSA history	28.8 (7.13–116.3)	< 0.001
Care dependency	6.1 (2.0–16.6)	0.001
Under cancer treatment	3.3 (1.1–9.4)	0.028

*CI* confidence interval, *OR* odds ratio.

### Stability assessment

The same three variables were shown to be the most stably selected parameters when the model building process underwent a bootstrapping evaluation. “MRSA history” was selected in almost 95% of runs, “care dependency ” in 52% and “under cancer treatment” in about 45%. Age was a relatively stable predictor as well, chosen in about 44% of runs (mutually exclusive to care dependency), followed by antibiotic treatment in the past 6 months with about 32%. The remaining variables were selected in less than 20% of the runs.

### Score building

A simplified score could be built including the same three variables. Each positive response to one of the three questions resulted in one point, so that score values between 0 and 3 could be reached. Since the probability cut-off of the regression model was extremely low at 0.01, there was no need to weight the score according to the regression coefficients. A single positive response to any of the three questions was enough to pass the probability threshold. When applying the score to the training dataset, 21.9% (263/1201) of all admitted patients had to be screened microbiologically to reach the reported diagnostic prediction accuracy.

### External validation

**Figure 1:**
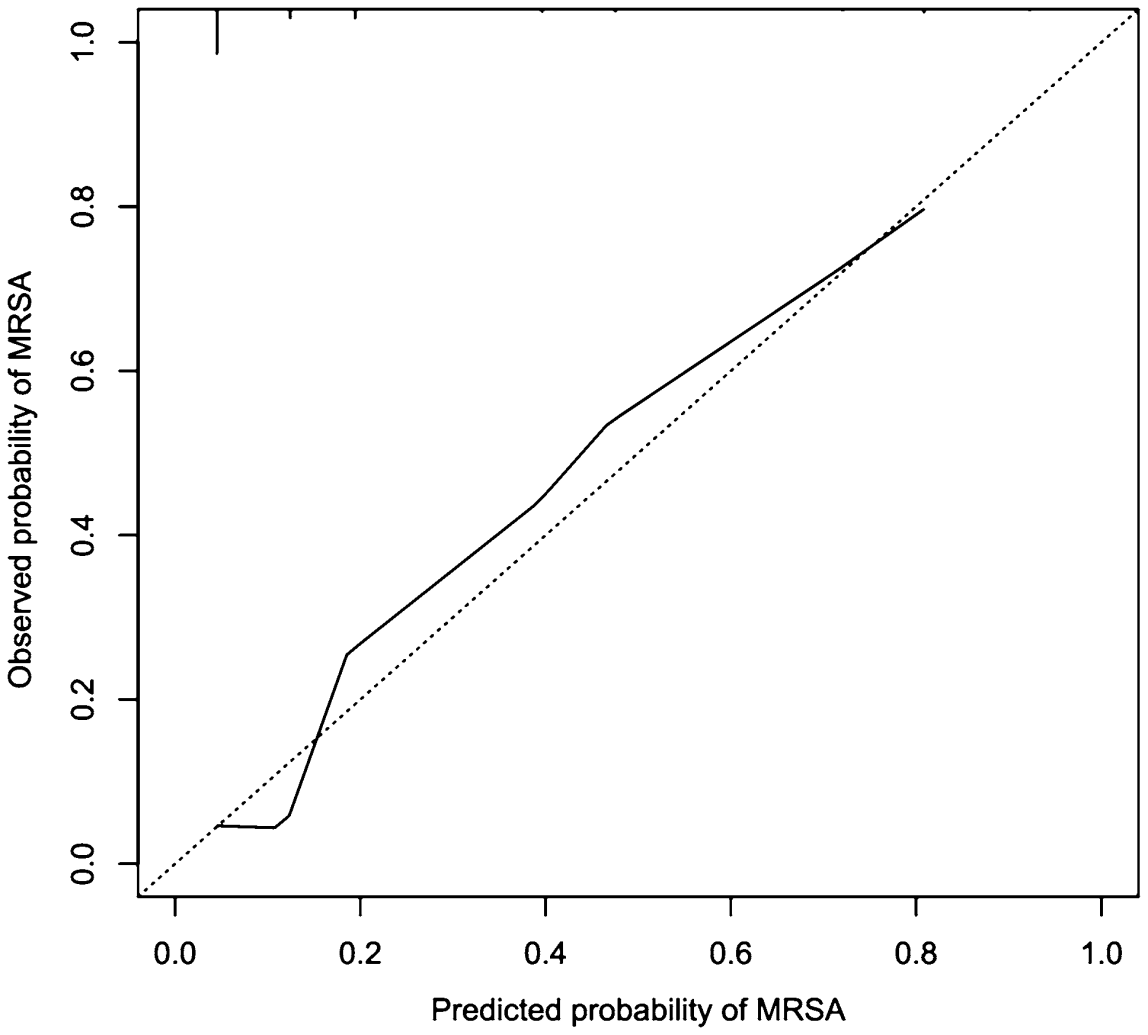
Calibration curve in the external validation dataset.

Applying the developed score to the validation dataset (Supplementary Table S1) resulted in a sensitivity of 78.4% (95% CI: 64.7–88.7%), and a specificity of 70.3% (95% CI: 65.0–75.2%); using the Bayes formula and an overall prevalence of 2% at admission, a positive predictive value of 5.1% (95% CI: 2.9–7.3%) and a negative predictive value of 99.4% (95% CI: 98.6–100%) could be calculated; 25.4% of the patients had to be screened to reach the diagnostic prediction accuracy described. Calibration was good over the whole range of predictions (Fig. 1). The calibration slope was 0.9 (95% CI: 0.7–1.1).

The developed model and the model based on the KRINKO risk factors resulted in similarly high sensitivities for the detection of MRSA carriers (Table 3). However, the specificity was lower for the derived KRINKO risk factors model so that the proportion of patients classified to be microbiologically screened was considerably higher when using KRINKO criteria than our proposed score (49.5% vs. 25.4%).

**Table 3:** Comparison of the developed score with the score based on the KRINKO risk factor list in the validation dataset.

	Sensitivity (95% CI)	Specificity (95% CI)	To be screened	AUC (95% CI)
Developed score	78.4% (64.7–88.7%)	70.3% (65.0–75.2%)	25.4%	0.82 (0.75–0.89)
KRINKO score	80.4% (66.9–90.2%)	41.8% (36.4–47.4%)	49.2%	0.61 (0.55, 0.67)

*AUC* area under the receiver operating characteristics curve, *CI* confidence interval.

*KRINKO* Commission for hospital hygiene and infection prevention.

### Sensitivity analyses

Univariable analysis of non-imputed data revealed only minor differences, and did not result in any qualitative change of results when compared to the primary analysis.

Because of the limited number of MRSA-positive participants in the training dataset, we additionally performed a model building process based on the validation dataset using the same procedure as described for the training dataset. Again, no major differences in the performance of the model on the validation dataset was observed.

## Discussion

We developed a model for individual prediction of MRSA carriage at the time of hospital admission for the region of Southeast Lower Saxony. The final model contained three predictors and could be easily transformed into a simple clinical score. In external validation, its diagnostic prediction accuracy was superior to a screening algorithm based on the KRINKO risk factor list when applied to our study region. Various sensitivity analyses provided evidence that the existing limitations of the underlying datasets did not affect overall results.

MRSA prevalence at hospital admission was 2.0% (95% CI: 1.5–2.7%) in our study. This is in line with other German studies that reported prevalence values between 1.6 and 2.3%^7, 14–16^. For the catchment area of the hospitals under study, MRSA prevalence in a population-based study was reported to be 1.3% (95% CI: 0.6–3.0%)^16^.

In univariable analyses, ten potentially predictive factors for MRSA carriage were found. They were either directly associated with MRSA carriage or related to increased age and morbidity. This is in line with other studies where a history of MRSA carriage was often the strongest predictor^17,18^. In addition, high age^19–21^ and parameters associated with “contact with healthcare”^22^, such as history of antibiotic therapy or inpatient treatment, presence of chronic diseases, living in a long-term care facility, dialysis or skin disease were regularly described as risk factors for MRSA carriage^19–23^.

The final predictive model included three parameters (MRSA history, care dependency, and being under cancer treatment). They were confirmed as the three most stable risk factors in the bootstrapping analysis. Age was the next important variable in our stability analysis. Increased age is a well-known predictor for MRSA carriage in many other studies^19–22^, which was also confirmed in the univariable analysis in our study. Despite the fact that age was included in many of the models in the stability analysis, we decided not to use age for the prediction model, because age and care dependency were selected mutually exclusive in the bootstrap runs and care dependency was the more stable predictor (and easier to include in simple clinical decision support tools) than the continuous variable age. Testing patients on geriatric wards might nevertheless be an alternative or additive screening concept, as increased MRSA colonization is common in this group^17, 18,24^.

A simple score with high diagnostic prediction accuracy was derived. Such a score is very easy to apply in clinical practice as information on all three risk factors usually is readily available at the time of admission. Thus, there is no need for access to additional data sources, as it has been the case for predictive models developed in other studies^19,21,22,25^.

Our score differs from others proposed, which often included a higher number of predictors and sometimes additional weighting factors, making implementation in admission settings and emergency rooms more difficult^18,22,26^. Harbarth et al.^22^ considered the high proportion of patients to be microbiologically tested in their study and the high logistical and financial costs to be a problem, and reduced the number of predictors in their model from nine to four. As a consequence, the proportion to be screened decreased from about 70% to 50%, while sensitivity decreased slightly from 86 to 84%. However, these results were confirmed only in an internal validation setting without external validation datasets.

Another study proposed an incremental risk score containing three unweighted factors (recent antibiotic treatment, intra-hospital transfer and inpatient treatment within the past 2 years), but excluded patients with known MRSA history and used the information on risk factors obtained from the electronic patient records^25^. For the classification rule of ≥ 1 risk factors present, the sensitivity was 87% in internal and 88% in external validation, with the number of patients to be screened as high as 70% and 58%, respectively; sensitivity declined dramatically to 61% and 44% when using two risk factors or more as the classification rule^25^.

Compared with a model based on the KRINKO risk factor list, our model and the derived score showed a similar sensitivity (78.4% vs. 80.4%), and higher specificity (70.3% vs. 41.8%), while the proportion to be screened was considerably lower (25.4% vs. 49.2%).

Two further studies examined the diagnostic prediction accuracy of screening based on KRINKO’s 2008 criteria^11^, and found a comparable predictive accuracy as in our study (sensitivity of 78.9% and 77.6%, and a proportion to be screened of 41.1% and 50.6%^7,18^).

Our score showed its cost-effectiveness by the considerably lower proportion of persons requiring microbiological screening at admission. Creamer et al.^26^ showed for their institution in Ireland as well that admission screening of MRSA-risk patients decreased costs by 60% compared to a form of screening where all patients were screened on admission. The ultimate aim of MRSA screening programmes is to decrease the incidence of hospital-associated MRSA infections. Reilly et al.^27^ showed in a Scottish study that MRSA screening can actually accomplish this.

### Limitations

Our study has several limitations, which correspond to the quality of data collection during routine clinical practice. A major limitation of our study was the number of MRSA-positive patients in the training dataset, which reduced the statistical power of the analyses. In total, only 1201 of 2556 patients could be included in the training dataset (47%), with the proportion of MRSA carriers being even lower at 38% (16 of 42). To assess if the low number of MRSA-positive cases in the training dataset might have affected model building, we performed a sensitivity analysis in which we used the validation dataset for training and vice versa; results were virtually unchanged. Due to the study design with patient-administered questionnaires, only few participants in intensive care units could be included. Since MRSA carriage is known to be higher there^2,6,28,29^, the screening recommendation might not be applicable to patients requiring intensive care support at admission; a screening recommendation might thus be extended to all patients admitted to intensive care units. The sampling schemes of the training and validation dataset were different. While the training dataset was derived using a classic surveillance setting with low MRSA prevalence, the validation dataset was designed in a more balanced way based on an ongoing universal screening program so that a larger number of MRSA-positive individuals could be included. This was done deliberately based on the experience with the training dataset but could have affected how the results of the study can be generalized. The model based on the KRINKO criteria did not include one of the criteria mentioned by KRINKO because it was not part of the questionnaire. Since the classification rule for the KRINKO model corresponded to at least one positive criterion, our analysis might have slightly underestimated the true sensitivity of the KRINKO model, while it would have overestimated specificity.

## Conclusions

We developed and externally validated a score for the identification of MRSA carriers at hospital admission in the region of Southeast Lower Saxony. The score showed better diagnostic prediction accuracy than the previous overall German screening considerations, with a lower proportion of individuals to be screened, and is easily applicable in clinical practice. The validity of the score outside the catchment area needs to be examined. Furthermore, it needs to be evaluated if additional universal screening of patients in intensive care units or geriatric patients leads to an improvement in sensitivity without disproportionately decreasing specificity.

## Supplementary information

Supplementary Information

## Data Availability

All data generated or analysed during this study are available from the corresponding author on reasonable request.
