# Supplementary material for: Development and external validation of a clinical prediction model for MRSA carriage at hospital admission in Southeast Lower Saxony, Germany
Source: Sci Rep. 2020 Oct 22;10:17998. doi: 10.1038/s41598-020-75094-6 (PMC7582828; doi:10.1038/s41598-020-75094-6)
Supplement: Supplementary file 1 — Supplementary Information [file 41598_2020_75094_MOESM1_ESM.pdf]

Supplementary information

Article title

Development and external validation of a clinical prediction model for MRSA carriage at hospital admission in Southeast Lower Saxony, Germany

Author names and affiliations

Gabriele Raschpichler, MD[1], Heike Raupach-Rosin, MD[1], Manas K. Akmatov, DrPH[1,2], Stefanie Castell, MD[1], Nicole Rübsamen, PhD[1,3], Birgit Feier, MD[4], Sebastian Szkopek, MD[5], Wilfried Bautsch, MD PhD[5], Rafael Mikolajczyk, MD[1,6,7], André Karch, MD[1,3,*]

1 Department of Epidemiology, Helmholtz Centre for Infection Research (HZI), Braunschweig, Germany.

2 Central Research Institute of Ambulatory Health Care in Germany (ZI), Berlin, Germany.

3 Institute for Epidemiology and Social Medicine, University of Münster, Münster, Germany.

4 Central Laboratory, Klinikum Wolfsburg, Wolfsburg, Germany.

5 Institute for Microbiology, Immunology und Hospital Hygiene, Städtisches Klinikum Braunschweig gGmbH, Braunschweig, Germany.

6 Institute for Medical Epidemiology, Biometry, and Informatics (IMEBI), Medical Faculty of the Martin Luther University Halle-Wittenberg, Halle (Saale), Germany.

7 Hanover Medical School, Hanover, Germany

Supplementary Table S1: Baseline characteristics of the study population (n=374) in the validation dataset, stratified by MRSA status

	Total		MRSA status				
			Positive		Negative		
	N	%	N	%	N	%	p value
	**374**	**100**	**51**	**13.6**	**323**	**86.4**	
Age [years]; *median (interquartile range)*	68.0 (19-100)		76.0 (19-92)		67.0 (19-100)		<0.001
Sex							0.222
• Male	189	51.1	22	43.1	167	52.4	
• Female	181	48.9	29	56.9	152	47.7	
• Missing	4		0		4		
Long-term care facility	29	7.8	12	23.5	17	5.3	<0.001
MRSA history	31	8.3	25	49.0	6	1.9	<0.001
MRSA in the household	14	3.7	8	15.7	6	1.9	<0.001
Diabetes mellitus	83	22.2	20	39.2	63	19.5	0.002
Diabetes treatment	74	19.8	17	33.3	57	17.7	0.009
Dialysis	7	1.9	2	3.9	5	1.6	0.245
Chronic skin disease	19	5.1	5	9.8	14	4.3	0.098
Under cancer treatment	62	16.6	5	9.8	57	17.7	0.162
Need for chronic care	77	20.6	27	52.9	50	15.5	<0.001
Open chronic wounds	31	8.3	6	11.8	25	7.7	0.333
Abscess / purulent skin disease	18	4.8	1	2.0	17	5.3	0.306
Outpatient treatment abroad past 12 months	4	1.1	0	0.0	4	1.2	0.424
Inpatient treatment past 12 months	189	50.5	34	66.7	155	48.0	0.013
Urinary catheter currently	54	14.4	12	23.5	42	13.0	0.047
Urinary catheter past 6 months	75	20.1	18	35.3	57	17.7	0.003
Antibiotics past 6 months	133	35.6	14	27.5	119	36.8	0.193
Occupational contact with livestock	10	2.7	1	2.0	9	2.8	0.734
Working in meat processing	0	0.0	0	0.0	0	0.0	—

The calculation of proportions does not include missing values in the denominator.
